# Changes in the Structure of the Bacterial Community Associated With *Ericaria amentacea* Blades Under Disturbed Conditions

**DOI:** 10.1002/pei3.70089

**Published:** 2025-10-08

**Authors:** Sarah Caronni, Lorenzo Federico, Pieraugusto Panzalis, Sara Villa, Sandra Citterio

**Affiliations:** ^1^ Department of Earth and Environmental Sciences University of Milano Bicocca Milano Italy; ^2^ Marine Protected Area of Tavolara Punta Coda di Cavallo Olbia Italy

**Keywords:** algae–bacteria interactions, *Cystoseira sensu lato*, health status, microbial communities

## Abstract

The macroalgae of the *Cystoseira sensu lato* complex host a great epiphytic bacterial community that significantly guarantees several important physiological processes, especially in case of disturbance. However, no direct evidence of relevant changes in the quali‐quantitative composition of these communities under anthropic disturbance is currently available. This work aims to characterize the epiphytic bacterial community associated with *Cystoseira sensu lato* populations in case of disturbance in Tavolara Punta Coda Cavallo Marine Protected Area (Sardinia, Italy). To this purpose, the abundance and health status of the most abundant species of the *Cystoseira* complex were evaluated in the three main islands of the MPA, characterized by different levels of protection. Moreover, thalli samples of the target species *Ericaria amentacea* (C. Agardh) Molinari and Guiry were collected in each zone to quali‐quantitative characterize its associated bacteria. Results confirmed a high abundance of *Cystoseira sensu lato* populations in the whole study area, with no chlorosis or damages related to the magnitude of disturbance. Significant differences were instead recorded in the quali‐quantitative characterization of the bacterial community among islands. Where the protection was lower, the abundance of living bacteria was higher and a relevant presence of bacteria involved in macroalgae resistance to disturbance, such as *Lutibacter* and *Psychromonas*, was observed. These results prove that the structure of the bacterial community associated with *E. amentacea* blades significantly changes in relation to disturbance. Moreover, they suggest that the good health status of these macroalgae observed also in cases of quite high disturbance could be related to a shift in its epiphytic bacterial community composition, that could, therefore, be actively involved in their adaptation.

## 1 Introduction

The macroalgae of the genus *Cystoseira sensu lato* complex (Fucales; Phaeophyceae) represent the dominant macroalgae in large areas of the intertidal coastal bedrock from the upper infralittoral to the upper circalittoral, where they form quite specific and peculiar biocenoses in association with *Padina* spp. and other macroalgae (Hereu et al. 2008). Such algae usually create dense, complex, and three‐dimensional canopies, commonly known as *Cystoseira* fringes, providing habitat, provisioning, and breeding grounds for many marine species (Giaccone and Bruni 1973). Indeed, they currently constitute one of the most productive and multifarious ecosystems of the Mediterranean Sea at the basis of several important services (Ballesteros 1988; Piazzi et al. 2018; Pinna et al. 2020).

In recent decades, *Cystoseira sensu lato* fringes have been depleted on a large scale in the Mediterranean Sea due to the impact of climate change (Lejeusne et al. 2010; Celis‐Plá et al. 2017; Verdura et al. 2021) and various anthropogenic factors, such as substratum loss caused by coastal activities, eutrophication, sedimentation, and pollution (Thibaut et al. 2005; Arévalo et al. 2007; Mangialajo, Chiantore, and Cattaneo‐Vietti 2008). This worrying condition has called for collective awareness of the serious threats that are affecting these macroalgae, and several studies focused on their maintenance and restoration have been carried out in recent times (e.g., de la Fuente et al. 2019; Eger et al. 2022; Smith et al. 2023; Mancuso et al. 2024). Upscaling for proper restoration, at least on a local scale, always requires an integrated approach, combining the ecological (phenology, population connectivity, and quantification of ecological indexes), historical (ubiquity in the territory and distribution patterns), and biological information available for the species of interest (Smith et al. 2023) with punctual data on its assemblages in the considered area. Therefore, in the last few years, a particular effort in acquiring specific information on the most relevant biotic and abiotic factors that mainly contribute to determining the health status of the macroalgae of the *Cystoseira sensu lato* complex has been made (Falace et al. 2010; Perkol‐Finkel and Airoldi 2010; Gianni and Mangialajo 2016; Eger et al. 2022). According to data currently available in literature, the structure and composition of the main bacterial communities associated with these species and, specifically, the epiphytic ones, should be considered among such factors (Zhou et al. 2016; Pedicini et al. 2023). Bacterial biofilms, indeed, appear to be fundamental for the survival of macroalgae, guaranteeing several different physiological processes, such as the absorption and release of nutrients and the production of secondary metabolites (Wahl et al. 2012). Macroalgal–bacterial associations are species‐specific and usually provide the algae with several benefits. Bacteria are known to modulate the defense of macroalgae, increasing their resistance to pathogens and their reproductive performance (Wahl et al. 2012). Therefore, they can remarkably influence their survival, reproduction, and growth (Menaa et al. 2020; Caronni et al. 2023), especially in disturbed conditions (Ghaderiardakani et al. 2020). Generally, macroalgae play an active role in modulating the richness and abundance of epiphytic communities (Mancuso et al. 2016), leading to the selection of bacterial strains promoting an increase in their tolerance to disturbed environmental conditions (Dobretsov and Qian 2002; Matsuo et al. 2005; Marshall et al. 2006; Spoerner et al. 2012; Wahl et al. 2012; Weigel et al. 2022). Such modulation is mainly driven by chemicals, such as phenolic compounds, antimicrobials, and antioxidants produced by the algae itself (Mannino and Micheli 2020; Abu‐Khudir et al. 2021).

On this matter, the macroalgae of the *Cystoseira sensu lato* complex are known to host a wide endophytic and epiphytic bacterial community associated with different parts of the alga, such as the thallus and the branches (Ghaderiardakani et al. 2020). However, the possible implication of such a community in determining the survival and growth ability of the algae as well as their health status is not yet clear, and the data currently available in the literature on the quali‐quantitative characterization of the epiphytic bacterial community associated with the *Cystoseira sensu lato* complex are still very scarce. Specifically, the paper by Mancuso et al. (2016) is almost the only one dealing with *Cystoseira sensu lato* complex epiphytic bacteria. The authors describe a quite low richness of epiphytic bacteria on young thalli of *Cystoseira compressa* (Esper) Gerloff and Nizamuddin in comparison with aged thalli, showing a gradual increase in bacterial richness. The observed trend was considered by the authors as the possible result of both a beneficial and a detrimental alga–bacteria interaction. Indeed, it could represent evidence of the bacterial infections that interest aged thalli, thus suggesting that the health status of this macroalga is negatively more than positively affected by bacteria. However, the obtained results can also be the result of a beneficial interaction, as already observed by Goecke et al. (2010) for other macroalgae. Specifically, the higher bacterial richness observed for *C. compressa* aged thalli could represent the result of the attempt of the macroalga to increase its resilient capacity during aging, shaping an ad‐hoc bacterial community, both in terms of composition and abundance. Beyond the above‐mentioned hypothesis, no certain information on the existence and on the eventual nature of a specific relationship between *Cystoseira sensu lato* macroalgae and their bacterial coatings is currently available. Therefore, further studies are needed to fill in this gap of knowledge, also considering the ecological importance of *Cystoseira sensu lato* assemblages in the Mediterranean Sea. In this perspective, the aim of this work was to characterize the epiphytic bacterial community of the macroalgae belonging to the *Cystoseira sensu lato* complex exposed to different levels of disturbance, investigating the existence of a relation between the quali‐quantitative composition of such a community and the health status of the macroalga. The tested hypothesis was that some relevant differences in the structure of epiphytic bacterial communities associated with *Cystoseira sensu lato* assemblages occurred in relation to the level of disturbance to which the algae were exposed, presumably affecting their health status.

To the purpose, *Cystoseira sensu lato* fringes present along the coasts of Tavolara Punta Coda Cavallo Marine Protected Area (Sardinia, Italy) were considered as a case study to acquire information on the structure and composition of epiphytic bacterial communities associated with their blades in different conditions of disturbance. A field‐laboratory measurement experiment was conducted in the MPA in the summer of 2023. According to a fully orthogonal sampling design, the quali‐quantitative composition of the whole macroalgal assemblage was, firstly, investigated in different areas of the MPA characterized by different levels of disturbance affecting benthic communities (e.g., Fraschetti 2006; Di Franco et al. 2009; Ceccherelli et al. 2011; Guidetti et al. 2006). Moreover, the health status of the whole *Cystoseira sensu lato* assemblages and of the most abundant species were assessed in each area. Finally, the microbiome associated with the blades of the most abundant and sensitive species belonging to the *Cystoseira sensu lato* complex in the area (*Ericaria amentacea*, see Section 3.1), which was considered as the key species of the work, was characterized both qualitatively and quantitatively in each zone.

## 2 Materials and Methods

### 2.1 Study Area, Experimental Design and Sampling Activities

**FIGURE 1:**
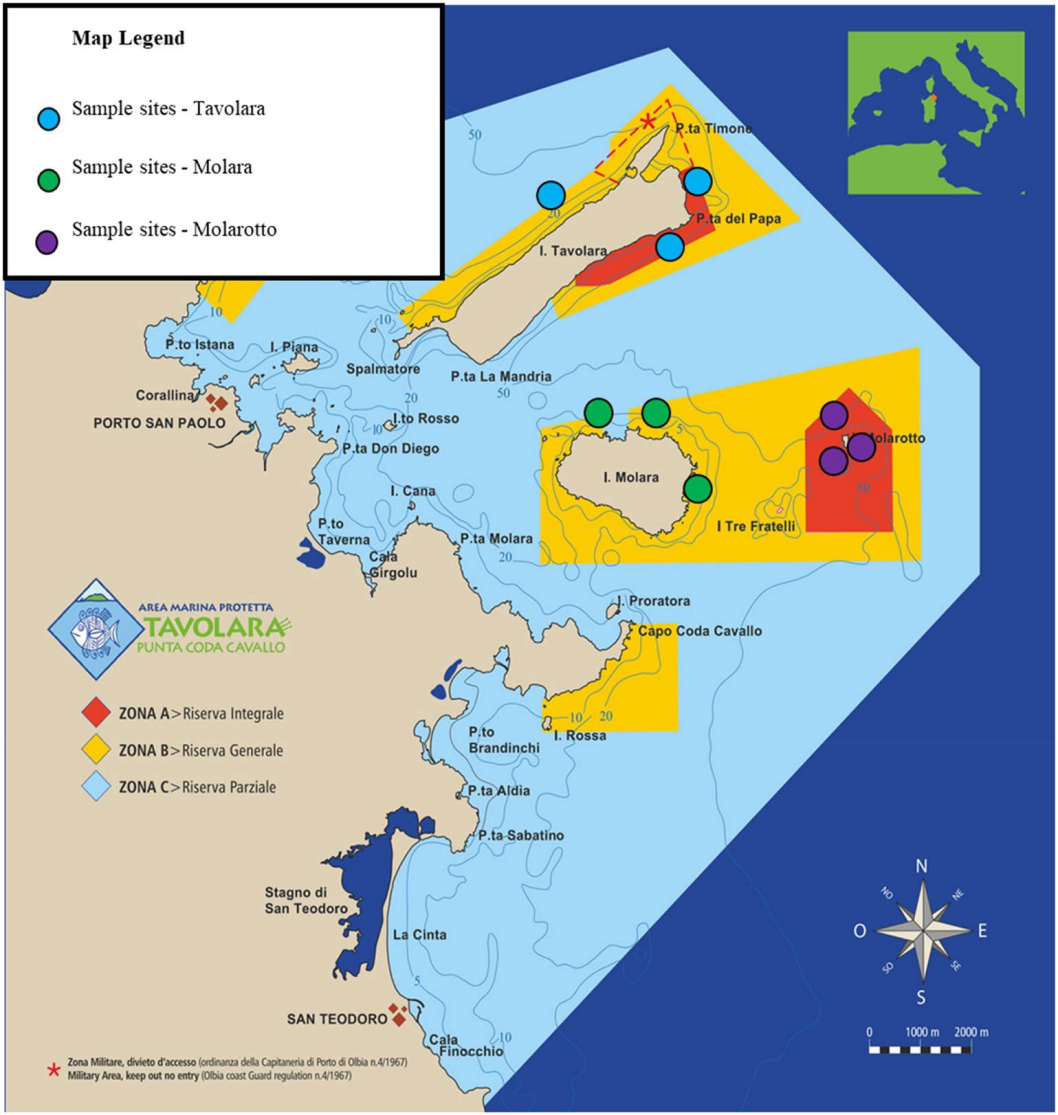
Cartographic map of Tavolara Punta Coda di Cavallo MPA (Sardinia, Italy). Zone A (red), Zone B (yellow) and Zone C (cyan) indicate marine coastlines characterized respectively by integral, general, and partial protected areas. *Source:* Official website of the MPA Tavolara Punta Coda Cavallo, Ministry of the Environment and Energy Security (MEES). Map legend: Blue points, Tavolara; green points, Molara, purple points, Molarotto.

The study was carried out in Tavolara Punta Coda Cavallo Marine Protected Area (Figure 1), along the North‐eastern coasts of Sardinia (Lat: 40° 54′ 22.32″ N, long: 9° 42″ 47.88″ E; Sardinia, Italy), where *Cystoseira sensu lato* fringes appear to be the most relevant macroalgal assemblages (Navone and Trainito 2008). In detail, the coasts of the three main islands of the MPA (Molarotto, Molara, and Tavolara Island) exposed different levels of disturbance due to the different protection regimes to which they are subject. On the whole, integral (A Zones), general (B Zones), and partial (C Zones) protection zones were considered. For each protection level, three sampling sites, differing for the substratum composition (limestone or granitic), the exposition (North, East, or South), the wave action (high or low hydrodynamic stress), the irradiation (high or low), and the coastal morphology (sheer vertical cliffs or low sloping coasts) were chosen in order to be sure to consider *Cystoseira sensu lato* populations in relation to all the different conditions characterizing each zone.

First of all, the species composition of the whole macroalgal assemblages of the three protection areas was assessed by visual census in transects of 50 m of length along the coast × 10 m of width (*n* = 3). In detail, 3 sub‐transects of 10 × 1m^2^ were defined for each transect and the total % cover of the substratum of each macroalga species was visually estimated in each, according to Prato et al. (2017), Rogers et al. (1994), and Mumby et al. (1997) with modifications. Specifically, four categories of percent cover were on the whole considered: I = <25%; II = 25%–50%; III = 50%–75%; IV => 75%. The macroalgae individuated in each transect were taxonomically identified at the species level, both directly in the field and in the laboratory, where some thalli for each different species were analyzed by optical microscopy, using the taxonomic keys proposed by ISPRA. In particular, for macroalgae belonging to the *Cystoseira sensu lato* complex Mangialajo, Sartoni, and Giovanardi (2008) was considered and the discrimination and recognition between species were carried out considering the presence or absence of phenological types, such as the presence of crespitous or non‐crespitous thalli, of tofuli, of spines or aerocysts, of spiny or smooth cauloids and of receptacles.

Moreover, the health status of *Cystoseira sensu lato* fringes was also evaluated in each transect applying an ad‐hoc modified version of the LBRHI index proposed by Blanfuné et al. (2023) to evaluate the health status of another important algal complex (*Lithophyllum* rims) (for *Cystoseira sensu lato* fringes the index acronym CyFHI—*Cystoseira sensu lato* fringes health index—was chosen for the index). Specifically, for each fringe different items were considered as descriptors: (i) presence of only living blades and entire thalli, visibly colored and not damaged; (ii) relevant presence of smooth and brown‐green colored substratum portions, covered by a thin algal layer corresponding to *Cystoseira sensu lato* recruitment of the year (reflecting a good health status); (iii) relevant presence of dead parts of blades and entire thalli, clearly discolored (white), eroded or anyway damaged; (iv) relevant presence of ephemeral green algae (*Ulvophyceae*); (v) relevant presence of unvegetated portions of substratum; (vi) (reflecting a bad health status). Basing upon the above‐mentioned descriptors, expressed as percent cover of surface area of the fringe, the index was calculated as the sum of the percentages of the categories indicating a good health status of the macroalgae of the *Cystoseira sensu lato* complex divided by the sum of the percentages of all the considered categories (Blanfuné et al. 2023).

Finally, several thalli of a key species belonging to the *Cystoseira* genus (the most abundant and sensitive one) were collected along each transect and used for the qualitative and quantitative characterization of the bacterial community associated with the algal blades.

### 2.2 Laboratory Work—Quali‐Quantitative Characterization of the Microbial Communities

#### 2.2.1 Sample Collection and Preparation

On the basis of data of the composition of the whole macroalgal assemblages in the study area (see Section 3.1) and of *Cystoseira sensu lato* complex ones, *Ericaria amentacea* (C. Agardh) Molinari and Guiry was defined as the target species to be considered for the characterization of the associated microbial community. Therefore, three thalli of the above‐mentioned macroalga were collected along each transect (one for each sub‐transect) and placed in plastic bags for their transport and storage. To remove non‐attached microorganisms, blades were treated according to the procedure suggested by Jensen et al. (1998), modified as follows. Each blade sample was rinsed on both sides with a 10 mL stream of filtered seawater, aseptically. The rinsed samples were placed in empty sterile petri dishes and vigorously swabbed on both sides with a sterile cotton‐tipped applicator. Swab tips were then placed in 15 mL micro centrifuge sterile tubes containing 0.5 mL of filtered seawater and vortex‐mixed for 30 s. Finally, the applicator tips were removed from the tubes and the resulting bacterial suspensions were vigorously hand‐shaken and diluted (1:4) with filtered seawater to obtain a final suspension volume of 2 mL.

#### 2.2.2 Bacterial Counts

Total bacterial counts (number of bacteria/cm^2^) were carried out by using epifluorescence microscopy with three different stains, according to standard procedures (Sgorbati et al. 1996). A first aliquot of the bacterial suspension was stained with filtered (0.2 μm) DAPI (1 μg/mL) for counting the total bacteria by visualization of nuclear, mitochondrial, and chloroplast DNA (Williamson and Fennell 1979). A second aliquot was stained with a mix of filtered SYBR green and propidium iodide to discriminate between alive and dead or anyway damaged bacteria (Eusébio et al. 2022). The bacteria were then observed with a Zeiss Axioplan microscope and a Bürker counting chamber was used to enumerate the bacteria. Counts were expressed as the number of bacteria for 1 cm^2^ of blade area.

#### 2.2.3 Bacterial DNA Extraction and Quantification

The extraction and quantification of epiphytic bacterial DNA was carried out according to the method reported in other studies (Pittino et al. 2018; Caronni et al. 2023). Total DNA was extracted from 1.5 mL of each suspension (kept at −20°C) with the FastDNA Spin for Soil kit (MP Biomedicals, Solon, OH, USA) by a solid nitrocellulose substrate, according to the manufacturer's instructions. Amplification of the V5–V6 hypervariable regions of the 16S rRNA gene for each sample was performed by PCR, for evaluating its quality on the original and on the 1:10 and 1:100 dilutions. A second PCR was therefore performed with GoTaq Green Master Mix (Promega Corporation, Madison, WI, USA) and 1 μM of each primer, to achieve a final volume of 2 × 50 μL for each sample. Customized oligonucleotide barcodes (6 bp) were added at the 5′ end. 783F and 1046R primers were therefore added, and the cycling conditions were initial denaturation at 94°C for 4 min, 28 cycles at 94°C for 50 s, 47°C for 30 s, 72°C for 45 s, and a final extension at 72°C for 5 min. The amplicons were purified using the Wizard SV Gel and PCR Clean‐up System (Promega Corporation, Madison, WI, USA) and quantified with Qubit (Life Technologies, Carlsbad, CA, USA). Libraries were prepared with nine samples each, identifiable thanks to different barcode pairs. Library preparation with the addition of standard Nextera indexes (Illumina Inc., San Diego, CA, USA) and sequencing with the MiSeq Illumina platform (Illumina Inc., San Diego, CA, USA), using a 2 × 300 bp paired‐end protocol, was performed. The obtained reads were demultiplexed according to the indexes and barcodes. The Uparse pipeline was used for the following elaborations. Forward and reverse reads were merged only if there were zero mismatches and quality filtered with default parameters. The Amplicon Sequence Variants (ASVs) were defined with an aggregative clustering of sequences with 97% of sequence identity using the DADA2 algorithm (Callahan et al. 2016). Suspected chimeras and singleton sequences were removed. ASVs classification at order and genus level was inferred using the RDP classifier. To compare diversity among samples that largely differed in the number of sequences, 20,000 sequences were randomly selected from each sample for which more than 20,000 sequences were available, normalizing to 20,000 sequences by resampling with repetition.

### 2.3 Data Processing and Analysis

Data regarding the percent cover of the substratum by *Cystoseira* species in the study area and those regarding the abundance of the bacterial community associated with *E. amentacea* were analyzed by means of univariate statistical analyses with the software GMAV 5. One‐way analysis of variances (ANOVAs) and Student–Newman–Keuls (SNK) tests were performed (Underwood and Chapman 1996). The composition of the bacterial community, expressed as Amplicon Sequence Variants (ASVs), was analyzed by means of multivariate statistical analysis with the software Past4.03, PermANOVA1.6, and PrimerV6 (PCA, PERMANOVA, SIMPER) (Anderson 2001; Legendre and Gallagher 2001). A more detailed description of the statistics is available in the Supporting Information: Section SM1.

## 3 Results

### 3.1 Abundance and Quality of *Cystoseira* Assemblages

Macroalgae belonging to the *Cystoseira* appeared to be the main components of macroalgal assemblages in all the transects of the three considered islands, with a cover between 50% and 75% (III category of cover) in all, while other macroalgae (mainly algal turfs of ephemeral green algae; *Padina pavonica* (L.) Thivy populations, *Laurencia obtusa* (Hudson) J. V. Lamouroux populations, and Corallinaceae assemblages including both erect and crustose species) were present with a cover lower than 25% (I category of cover). The remaining portions of the substratum appeared, instead, to be unvegetated and bare rocks were clearly visible.

**FIGURE 2:**
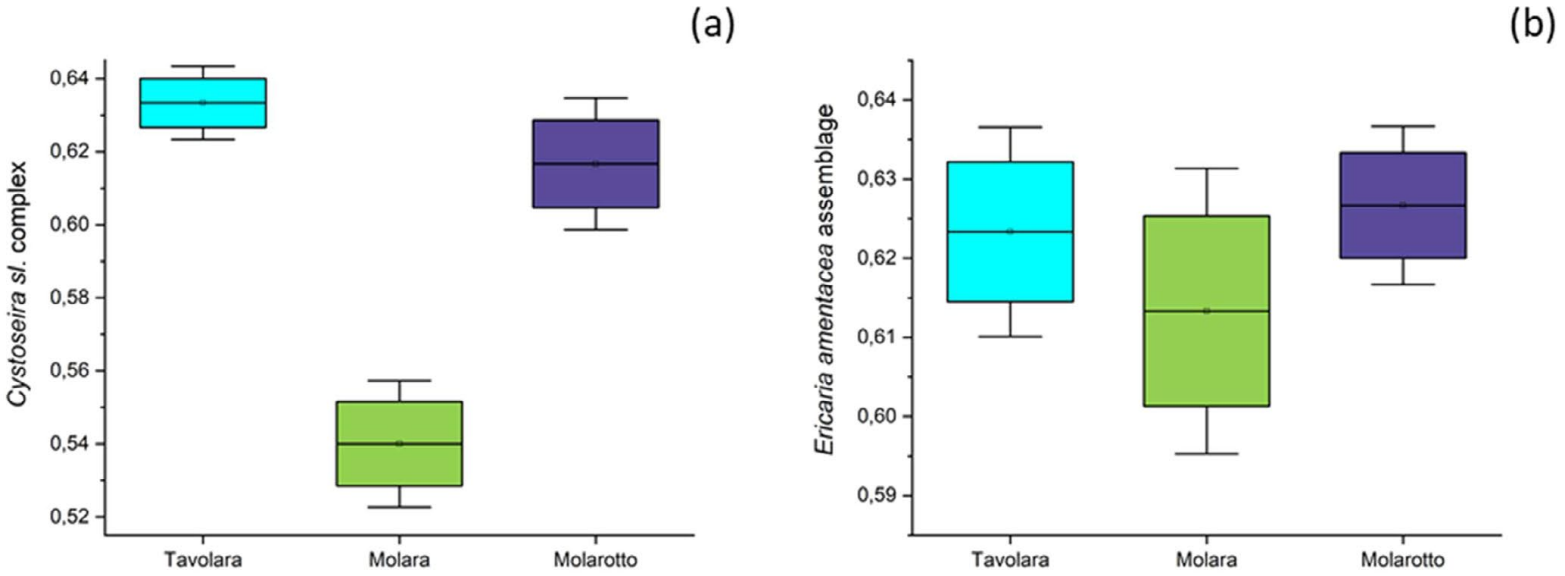
Health status of *Cystoseira sensu lato* (a) and *E. amentacea* (b) assemblages for all the sampling sites and transects of MPA according to the applied index.

With regard to the health status of *Cystoseira* assemblages, it appeared high (with values of the CyFHI index higher than 0.6) or at least good (with values of the CyFHI index higher than 0.5) in all the sampling sites, where a low number of damaged blades and only a few evidences of chlorosis were detected, especially for the most abundant *Cystoseira* species in all three transects, that appeared to be *E. amentacea* (*F*_(1,2)_ = 0.45; *p* = 0.042), for which, moreover, no relevant differences in the thallus morphology were overall detected among sampling sites (Figure 2).

**FIGURE 3:**
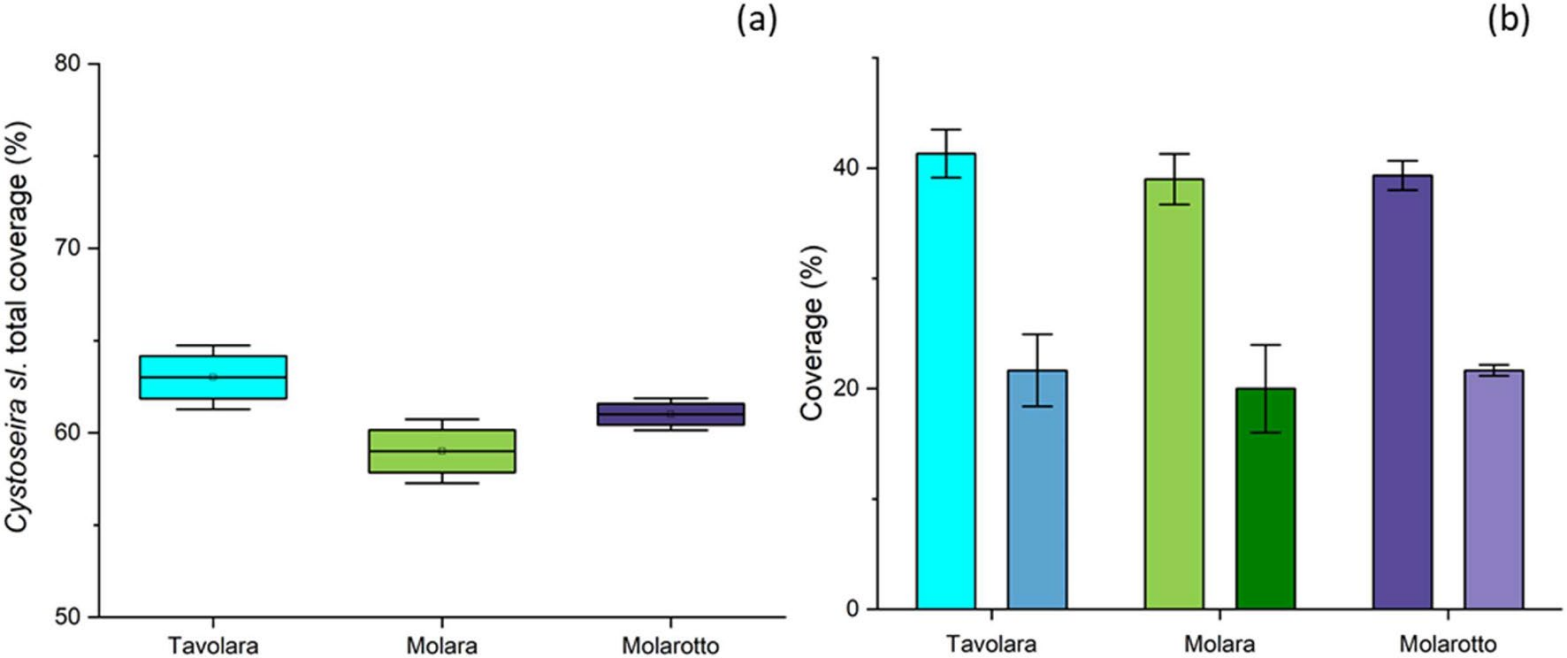
Mean total percent cover (±SE) of the whole *Cystoseira* assemblage (a) and of *E. amentacea* (histograms from the left of sample sites) in comparison to the other *Cystoseira species* (histograms from the right of sample sites) (b) along the coasts of the three different islands of the MPA.

Both referring to the total percent cover of *Cystoseira* genus (Figure 3a) and to that of *E. amentacea* (Figure 3a,b), no statistical differences were recorded among the three islands (Tables S1 and S2). Nevertheless, a higher cover was on the whole observed along the coasts of Tavolara, while Molara exhibited the lowest percent cover (Figure 3a,b).

### 3.2 Characterization of the Bacterial Community

**FIGURE 4:**
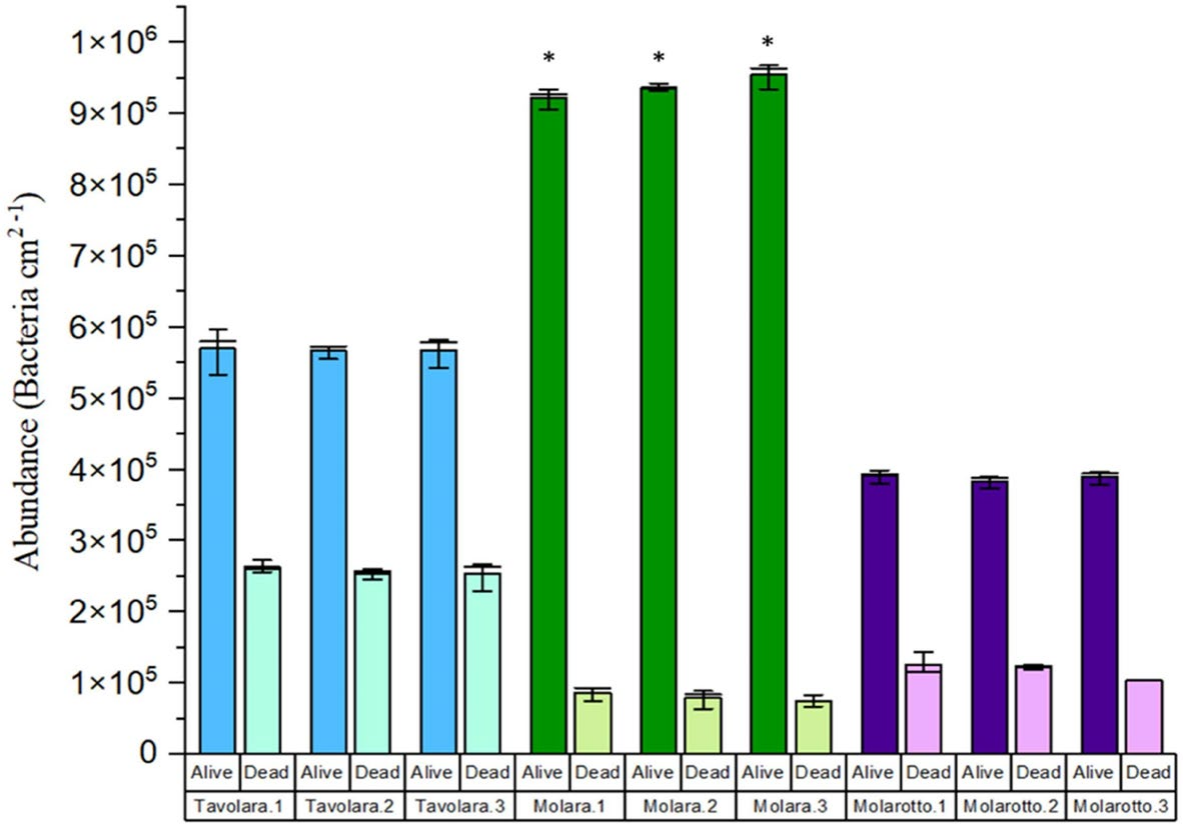
Mean abundance of alive and dead bacteria (±SE) associated with *E. amentacea*, among the transects (1, 2, and 3) of Molara, Molarotto, and Tavolara islands. Asterisks denote significant differences (0.01 ‘*’ 0.05).

With regard to bacterial abundance on the blades of *E. amentacea*, a higher number of living bacteria compared to dead ones (mean values of 412,635 ± 37,969; 509,058 ± 104,013; and 252,879 ± 32,936 for Tavolara, Molara, and Molarotto, respectively) was observed in all the transects across the islands (Figure 3). Moreover, some relevant differences in the number of alive and dead bacteria were recorded among Molara, Molarotto, and Tavolara Islands (Figure 4). Particularly, the number of alive bacteria was notably higher in Molara (ranging between 934,099.58 ± 160,417.95 and 526,489.47 ± 244,388.87 alive‐dead bacteria/cm^2^), compared to Molarotto (between 399,063.88 ± 42,036.94 and 290,981.98 ± 6081.70 alive‐dead bacteria/cm^2^) and Tavolara (between 379,870.13 ± 12,586.01 and 147,308.59 ± 28,917.54 alive‐dead bacteria/cm^2^) (Table S3).

**FIGURE 5:**
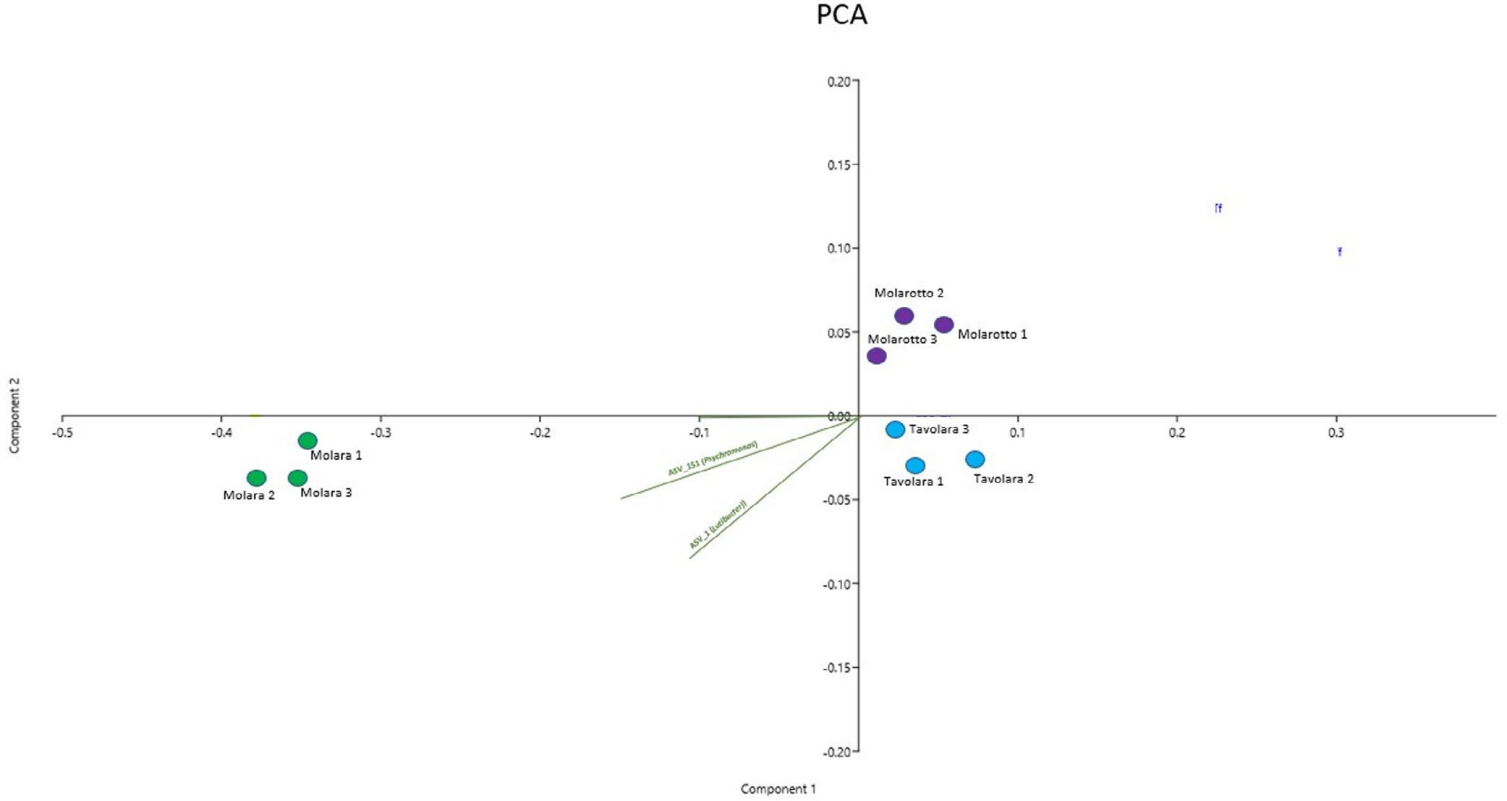
PCA biplot on Hellinger‐transformed abundances of each ASV for the experimental samples. The plot also shows the principal bacterial taxa that mostly contribute to the patterns observed. Symbol colors are used for the different study areas: Green dots stand Molara; purple dots stand for Molarotto; red dots stand for Tavolara.

**FIGURE 6:**
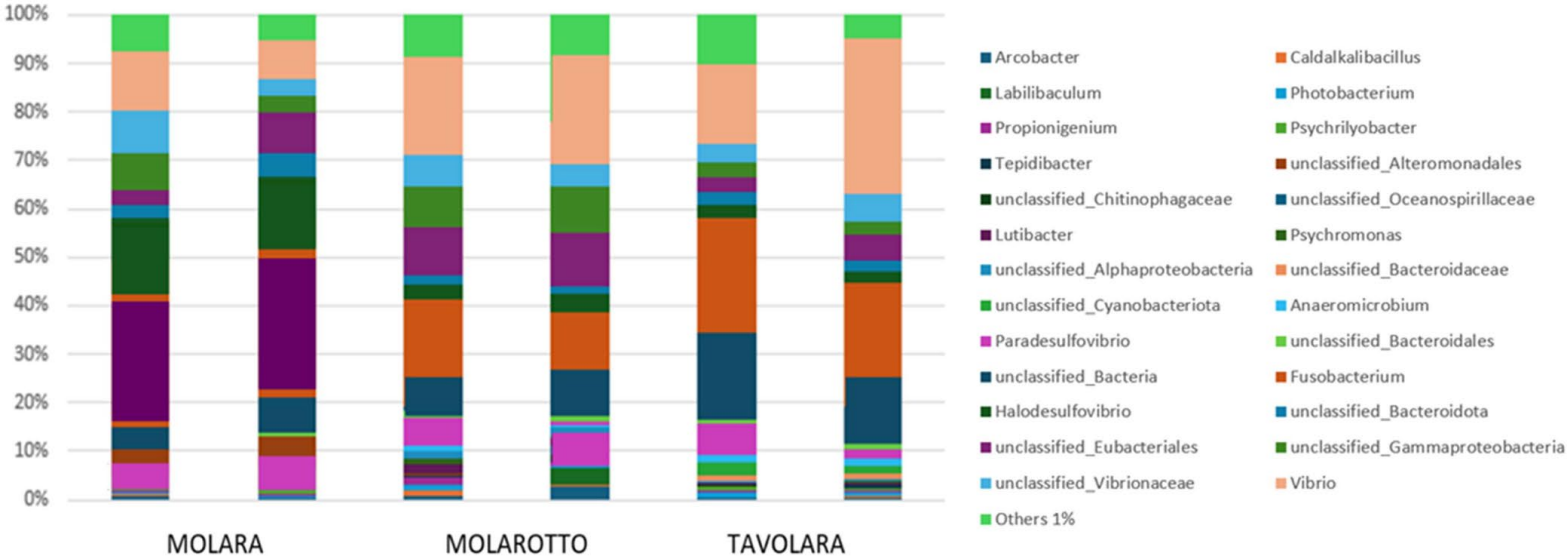
Overall composition of the epiphytic bacterial community associated with *E. amentacea*, analyzed for each sampling site corresponding to the different protection levels of the MPA. For each genus, the relative abundance is indicated as a percentage of the total genera. In the “others” category, all genera with an abundance less than 1% are grouped together.

Concerning the qualitative characterization of the epiphytic bacterial communities associated with *E. amentacea* blades, more than 127 AVSs were observed in the considered samples. As highlighted by the MDS (Figure 5), some relevant differences in the composition of the bacterial community were highlighted among the three different islands (Figure 6), as emerged also from the PERMANOVA (*p* = 0.041). Specifically, the results of the pairwise post‐PERMANOVA test underlined that the Molara epiphytic bacterial community resulted to be significantly different from that of the other islands. Indeed, the SIMPER test showed a dissimilarity of 68% between the epiphytic assemblages of Molara and Molarotto islands and of 65% between Molara and Tavolara islands, while microbial assemblages between Molarotto and Tavolara islands showed the lowest dissimilarity (32%). In detail, analyzing the taxa composition of such communities, the genera *Lutibacter* sp. (4.89%) and *Psychromonas* sp. (4.51%) emerged as the most abundant taxa on blades collected along the coasts of Molara Island, differently from Molarotto and Tavolara where they appear not to be so represented (Table S4).

## 4 Discussion

This work provides new insights on the composition of the epiphytic bacterial community associated with the macroalgae of the *Cystoseira sensu lato* complex and on the structural changes they can undergo in case of disturbance.

First of all, analyzing data regarding the percent cover of the substratum of either the whole *Cystoseira sensu lato* assemblage of Tavolara Punta Coda Cavallo MPA or of the key species *E. amentacea*, no significant differences were recorded among islands characterized by different levels of protection and therefore by different disturbances. Indeed, a slow reduction only occurred in Molara, where C protection zones with low protection rules were considered, but such decrease appeared not to be significant. Moreover, in all the sampling areas, a high or anyway good quality status was described for *Cystoseira* assemblages, with the highly sensible species *E. amentacea being* dominant not only in A and B protection zones but also in the C ones, where the level of protection is lower. Usually, C zones of marine protected areas represent buffer zones between the areas of greatest naturalistic value subject to high protection and the not protected areas surrounding the MPA (Smith et al. 2023). Sure enough, in the latter, anthropogenic pressures and human activities are expected to be greater and to directly influence the more exposed communities. As already stated, indeed, different levels of disturbance affecting the benthic communities of the three areas of the MPA and impacting especially the more sensitive species have already been extensively described (e.g., Fraschetti 2006; Di Franco et al. 2009; Ceccherelli et al. 2011; Guidetti et al. 2006). According to literature, *Cystoseira sensu lato* assemblages and, specifically, those composed mainly of *E. amentacea*, appear to be among them, as the great part of *Cystoseira sensu lato* macroalgae appear to be quite sensitive to environmental and anthropic pressures, which usually seriously stress them (Soltan et al. 2001; Bulleri et al. 2002; Falace et al. 2010). In case of high disturbance, therefore, a gradual reduction of the abundance of the macroalgae belonging to the *Cystoseira sensu lato* complex, and especially of the more sensitive species, like *E. amentacea*, occurs. In such conditions, moreover, also a relevant worsening of their health status is described, thus causing their replacement with other more tolerant species (Barcelo 2023). The magnitude of such changes is expected to be proportional to that of the disturbance (Alestra and Schiel 2015). In this perspective, the quite similar percent cover of the substratum of *Cystoseira sensu lato* macroalgae and especially of *E. amentacea* observed along the coasts of the three islands, despite the different levels of protection, appears as a quite unexpected result. Indeed, even if in the C zones of the MPA the level of disturbance is on the whole limited if compared with that of fully not protected areas (Di Franco et al. 2009), *Cystoseira sensu lato* macroalgae seem to be characterized by a greater tolerance and a higher resilient capacity than previously estimated (Thibaut et al. 2014). According to the information available in literature, the great part of the macroalgae belonging to the *Cystoseira sensu lato* complex seem to have limited tolerance in case of disturbance, contrarily to what usually observed for algae, the majority of which are characterized by a quite high phenotypic plasticity that enables them to thrive in quite unfavorable conditions (Morales and Trainor 1997; Lürling 2003). This seems to be mainly due to some specific and peculiar traits that seem to disadvantage *Cystoseira sensu lato* macroalgae in terms of resistance. Beyond the low rate of nitrogen and phosphorous uptake of these algae (Pedersen and Borum 1997; Lotze and Schramm 2000), for some *Cystoseira sensu lato* species among which *E. amentacea*, the quite low phenotypic plasticity is among the main factors leading to an increased sensitivity to several of the anthropogenic disturbances normally present in the C zones of MPAs (Graiff et al. 2015; Schmid et al. 2021). For example, *E. amentacea*, as well as other microalgae of the same taxonomic group, displays a marked negative buoyancy, due to which it is usually confined to shallow rocky areas, where it is highly exposed to wave effects (Thibaut et al. 2014), that significantly increase where recreational boat traffic is heavy, as in C zones. Therefore, it is possible to speculate that other mechanisms than those related to algae physiology are implicated in determining the distribution of the macroalgae belonging to the *Cystoseira sensu lato* complex in the MPA, independently from protection. Specifically, coastal morphology, irradiance, and exposure must be considered taking into account that they can differ among sites with the same levels of protection.

Also, analyzing in detail, the results of the quantitative and qualitative characterization of epiphytic communities associated with the key species of this study, some other interesting information can be obtained. First of all, a significantly higher number of alive bacteria was observed on *E. amentacea* blades in Molara compared to that observed for Molarotto and Tavolara samples. Moreover, also considering the taxa composition of such communities, some relevant differences were observed between Molara, where the protection is lower, and the other two islands, subjected to a higher level of protection.

As the same macroalgal species (*E. amentacea*) was investigated in all the areas and no relevant differences in the morphology of its thalli were detected among them, it is plausible to exclude that the observed dissimilarities in the composition of bacterial communities associated with *Cystoseira* are related to the complexity of the algal thallus, currently considered one of the key factors in defining the structure of bacterial communities associated with macroalgae (Mancuso et al. 2016), although specific phenological analyses were not carried out. Furthermore, as already stated, a good health status and a high abundance of these algae were recorded in all the sampled areas, confirming that the lower abundance of bacteria observed in Tavolara and Molarotto was not the result of the presence of a reduced colonizable area related to the presence of damaged or anyway injured blades, as already observed for other epiphytes (Beckett et al. 1992). On the other hand, the good health status of *Cystoseira* assemblages observed in Molara suggests that the higher bacterial abundance recorded along the coasts of this island is not due to an increased bacterial colonization of diseased thalli, as described by Fernandes et al. (2012) for the red macroalga *Delisea pulchra* (Greville) Montagne.

On this matter, it is possible to speculate that the relevant quali‐quantitative differences in the composition of the epiphytic bacterial community associated with *E. amentacea* blades in relation to the protection level and, therefore, to the magnitude of disturbance could be involved in the response of these macroalgae in case of disturbance. This hypothesis has already been proposed also by Mancuso et al. (2016) and Selvarajan et al. (2019) in other similar studies. Indeed, it is well known that microbial colonization of the plant surfaces is a dynamic process, strictly related to environmental conditions (Rao et al. 2006). At this regard, Saha and Weinberger (2019) postulated for the first time that aquatic plants are able to chemically “garden” protective microorganisms to strengthen their resistance in case of disturbance. In this perspective, it is plausible to speculate that in the C zones of the MPA, subject to partial levels of protection and, therefore, potentially more disturbed, *E. amentacea* populations could have modulated their associated bacterial community to act defense mechanisms helping them to achieve a higher resilience than that allowed by their physiology. Actually, the existence of a direct relation between bacterial modulation and algae health status has already been observed for other species, like the ones belonging to the *Caulerpa* genus (Caronni et al. 2023), for which the presence of toxic compounds induced the species to actively respond to oxidative stress with the production of substances that favored the development of a rich and specific bacterial community, involved in its resistance and in toxic compounds degradation. Also, Malik et al. (2020) emphasized that macroalgae have the capability to secrete various organic substances that facilitate the multiplication of bacteria and contribute to the formation of specific microbial biofilms on the macroalgal thallus surface and, especially, on blades. Due to their fast growth, indeed, bacteria provide a quite rapid response to disturbance, forming in a short time an efficient epiphytic protective layer (Menaa et al. 2020). Then, it is possible to hypothesize that, in case of disturbance, a major secretion of these compounds can occur in order to facilitate specific bacteria proliferation and, therefore, algal survival (Egan et al. 2013). Accordingly, the presence of a structured and rich epiphytic community on potentially disturbed macroalgae seems to be mediated by info‐chemical processes that depend mainly on the conditions to which the algae are exposed (Menaa et al. 2020; Caronni et al. 2023). In this perspective, focusing on the results obtained for *E. amentacea* bacterial community, it is plausible to speculate that an enhanced production of antimicrobial substances and phytochemicals occurs in the C zones of the MPA, where disturbances are more relevant, thus favoring the selection of those bacterial taxa that can allow them to maintain an ecological optimum despite the adverse conditions.

Also, the results of the qualitative characterization of *E. amentacea* associate bacterial community seem to support this hypothesis. As a matter of fact, low protection zones showed a higher abundance of some bacterial taxa that have already been described as involved in macroalgae resistance in case of disturbance, in comparison to the other islands. Indeed, bacteria belonging to the genera, *Lutibacter* sp. and *Psychromonas* sp. appeared to be particularly abundant on *E. amentacea* blades collected in Molara. With regard to bacteria of the genus *Lutibacter* sp., belonging to the family Flavobacteriaceae, they have already been observed on the blades of other macroalgae belonging to the *Ulva* and *Laurencia* genera, for which they appeared to be among the most abundant taxa, actively involved in growth and morphogenesis, especially in case of environmental disturbance (Gu et al. 2023; Ghaderiardakani et al. 2024). Moreover, the Flavobacteriaceae family is known to include several taxa playing an important role in the degradation of organic matter and in the functioning of biogeochemical cycles (Kirchman 2002). Consequently, it is possible to speculate that the increase of flavobacteria such as *Lutibacter* sp. on the blades of the algae collected in the C zone of the MPA, where organic matter and nutrients are more abundant, may represent a protection against eutrophication and organic xenobiotic contamination, as already speculated by Goecke et al. (2010) for other species.

Furthermore, the ASVs associated with the genus *Psychromonas* appeared to be particularly abundant in Molara samples. According to Katsuhiro et al. (2024), the abundance of bacteria belonging to this genus is directly related to the number of alginates produced by algae. Alginates are among the most abundant secondary metabolites produced by stressed or otherwise disturbed algae, among which those of the *Cystoseira* genus (Messina et al. 2019). Furthermore, the presence of bacteria of the genus *Psychromonas* has already been described for other algae exposed to disturbed conditions (Lee et al. 2006), even if no detailed information on their mechanisms of action is available. Therefore, the presence and abundance of *Psychromonas* on *E. amentacea* could be driven by chemicals, as suggested by Mannino and Micheli (2020), for other algae–bacteria interactions.

In conclusion, the obtained results proved that some relevant differences in the structure of the bacterial community associated with *E. amentacea* blades occur in the case of disturbance. On this matter, it is likely possible to hypothesize that the high abundance and the good health status observed for such macroalgae also in the C zone of the MPA (Molara) can be, at least partially, related to a shift in the taxa composition of its epiphytic bacterial community that could be involved in the seaweed adaptation to disturbance. However, more focused and detailed studies must be performed to verify this hypothesis.

## Conflicts of Interest

The authors declare no conflicts of interest.

## Supporting information


**Appendix S1:** pei370089‐sup‐0001‐AppendixS1.docx.

## Data Availability

The data that support the findings of this study are openly available in ZENODO at https://doi.org/10.5281/zenodo.17150539.
