# Supplementary material for: Changes in the Structure of the Bacterial Community Associated With Ericaria amentacea Blades Under Disturbed Conditions
Source: Plant Environ Interact. 2025 Oct 8;6(5):e70089. doi: 10.1002/pei3.70089 (PMC12508260; doi:10.1002/pei3.70089)
Supplement: Supplementary file 1 — Appendix S1: pei370089‐sup‐0001‐AppendixS1.docx. [file PEI3-6-e70089-s001.docx]

**The structure of the bacterial community associated to *E. amentacea* blades changes under disturbed conditions**

Sarah Caronni^1^, Lorenzo Federico^1^*, Pieraugusto Panzalis^2^, Sara Villa^1^, Sandra Citterio^1^

^1^University of Milano Bicocca, Piazza della Scienza 1, 20126, Milano, Italy

^2^Marine Protected Area of Tavolara Punta Coda di Cavallo, Via Dante 1, Olbia, Italy

*corresponding author: [lorenzo.federico@unimib.it](mailto:lorenzo.federico@unimib.it)

**SUPPLEMENTARY MATERIALS No.1 (SM1)**

**Detail of the performed univariate and multivariate statistical analysis**

**Univariate Statistical Analysis**. Univariate analysis were performed to test for differences in the total percentage cover of the substratum of *Cystoseira sp.* (one-way ANOVA: Protection, 3 levels, fixed), in the abundance of the key species *E. amentacea* (two-way ANOVA: Protection, 3 levels fixed: A *vs* B *vs* C; Species, 2 levels, fix: *E. amentacea* vs other *Cystoseira* species) and in the abundance and health of the bacterial community associated to the key species *Ericaria amentacea* (two-way ANOVA: Protection, 3 levels fixed: A *vs* B *vs* C; State, 2 levels, fixed*:* Dead *vs* Alive). Cochran’s tests were run prior to each ANOVA to test for homogeneity of data variances and normality was assured by Kolmogorov-Smirnov test. One-Way ANOVAs test was performed for testing differences between any treatments using mean values of the data. Student–Newman–Keuls (SNK) *post hoc* tests were used for comparison in case of significant ANOVA results, testing all the possible pairs of means computed on the considered groups (Underwood, 1997).

**Multivariate Statistical analysis**. Multivariate analyses were based on Hellinger distance, which depends on the differences in OTU proportion between samples, decreases the importance of ASV abundance over their occurrence and avoids the double-zero problem when comparing ASV composition between samples (Legendre and Gallagher, 2001; De Cáceres and others, 2010). A Principal Component Analysis (PCA) was performed on all samples to visualize data distribution. A distance-based permutational multivariate analysis of variance (PERMANOVA) (Anderson, 2001) was then performed to test for differences in the genus composition of the bacterial community of *E. amentacea* among the three islands of Tavolara Punta Coda di Cavallo MPA. The analyses were based on Bray-Curtis dissimilarities calculated on normalized data (Hellinger). Each term in the analysis was tested using 9999 random permutations.

**SUPPLEMENTARY MATERIALS No. 2 (SM2)**

**Table S1.** Statistical results referred to the *Cystoseira* genus total percentage cover (One-Way ANOVA, SNK post hoc test). Values for P < 0.05 were considered statistically significant.

| Source | DF | F | P |
| --- | --- | --- | --- |
| Level of Protection | 2 | 1,76 | 0,2504 |
| RES | 6 |  |  |
| TOT | 8 |  |  |
| Cochran's Test | | | |
| C = 0.5176 (Not Significant) | | | |

**Table S2.** Statistical results referred to the abundances of *Ericaria amentacea* (A) compared to the others *Cystoseira* genus (B) total percentage cover (Two-Ways ANOVA, SNK post hoc test). Values for P < 0.05 were considered statistically significant. Asterisks denote significant differences to control (Signif. codes: 0 ‘***’ 0.001 ‘**’ 0.01 ‘*’ 0.05).

| Source | DF | F | P | SNK |
| --- | --- | --- | --- | --- |
| Level of Protection | 2 | 0,45 | 0,67 |  |
| Species | 1 | 0,45 | **0,042*** | A > B |
| Protection vs Species | 2 | 0,29 | 0,75 |  |
| RES | 12 |  | |  |
| TOT | 17 |  |  |  |
| Cochran's Test | | | |  |
| C = 0.5176 (Not Significant) | | | |  |

**SUPPLEMENTARY MATERIALS N°3**

**Table S3.** Statistical results referred to the abundance of alive and death bacteria (± SE) associated with *Ericaria amentacea* collected from Molara, Molarotto and Tavolara islands (Two-Ways ANOVA, SNK post hoc test). Values for P < 0.05 were considered statistically significant. Asterisks denote significant differences (Signif. codes: 0 ‘***’ 0.001 ‘**’ 0.01 ‘*’ 0.05).

| Source | DF | F | P |
| --- | --- | --- | --- |
| Level of Protection | 2 | 400,01 | **0,000***** |
| State (Alive vs Dead) | 1 | 4620,38 | **0,000***** |
| Protection vs State | 2 | 593,35 | **0,000***** |
| RES | 18 |  | |
| TOT | 17 |  |  |
| Cochran's Test | | | |
| C = 0.3412 (Not Significant) | | | |

**SUPPLEMENTARY MATERIALS N°4**

**PERMANOVA**

**Permutational MANOVA**

**Table S4.** Statistical results referred to the Permutational MANOVA (PERMANOVA) referred to the composition of the epiphytic bacterial communities associated with *Ericaria amentacea*.

| PERMANOVA | | | | | | | |
| --- | --- | --- | --- | --- | --- | --- | --- |
| ISLAND | | | | | | | |
| Source | df | SS | MS | Pseudo-F | P(perm) | perms | P(MC) |
| Level of Protection | 2 | 8036,2 | 4018,1 | 13.212 | 0,0407 | 15 | 0,3119 |
| RES | 3 | 9124,1 | 3041,4 |  |  |  |  |
| Total | 5 | 17160 |  |  |  |  |  |

**References**

Anderson, M. J. (2001). A new method for non‐parametric multivariate analysis of variance. Austral ecology, 26(1), 32-46.

De Cáceres, M., Legendre, P., Moretti, M. (2010). Improving indicator species analysis by combining groups of sites. Oikos, 119(10), 1674-1684.

Legendre, P., Gallagher, E. D. (2001). Ecologically meaningful transformations for ordination of species data. Oecologia, 129, 271-280.

Underwood, A. J. (1997). Experiments in ecology: their logical design and interpretation using analysis of variance. Cambridge university press.
